# Supplementary material for: Supplementing dairy feed by dicalcium phosphate and effect on dry matter intake, digestibility, milk composition, and blood mineral balances in crossbred dairy cows
Source: PLoS One. 2023 Nov 17;18(11):e0282879. doi: 10.1371/journal.pone.0282879 (PMC10655975; doi:10.1371/journal.pone.0282879)
Supplement: Attachment — Submitted filename: resopns to editors and reviewers comment.docx [file pone.0282879.s002.docx]

**Response to editor's for reviewers' comments**

# Title: Effect of Dicalcium Phosphate Supplementation on Feed Intake, Milk Composition, and Mineral Balances in Crossbred of Dairy Cows

**Ref.:  Revision required [PONE-D-22-28223]**

Journal: PLOS ONE

First of all we would like to thank and appreciate the reviewers for their valuable comments and suggestions it will address very well and would significantly improve the quality of our manuscript.

We have accepted most of the suggestions forwarded by the Reviewers and corrected the manuscript accordingly. Where we had a different idea to the reviewers' comments we have kindly tried to explain our point.

Specific responses for specific issues raised by the Reviewers are given below (in blue).

**Comments from the editors and reviewers:**
1. **Reviewer 1.**

- Most of the issue which raised by the first reviewer was comments about the modification of title, number of animals in each treatment, about the parity of animals, repeated words and sentences such as; Table 1, 2 and 3 are not important b/s they show the composition and or deficiency in the previous study of mineral not the level of supplementation for each treatment, instead referring the total deficiency of those minerals in the single sentence and indicating the source is enough. ANOVA, GLM, SAS
- We have accepted and change comments in in the title, Abstract, title, methodology result and discussion part and conclusion

**A**. About title we modified title:” Supplementing Dairy Feed by Dicalcium Phosphate and Effect on Dry Matter Intake, Digestibility, Milk Composition, and Blood Mineral Balances in Crossbred Dairy Cows “

**B**. About abstract, methodology, result and discussion as well as conclusion and recommendations we accepted the comment and we go through and modified, corrected which are found in manuscript of response to reviewers.

- However, one of the comment raised by the first reviewer’s was about experimental animals: we were a total of eight mid- lactating cows 106.0 ± 5.0 day (mean ± SD) lactation stage, with one to four parities of cross breed of Holstein Friesian X F1 Boran cows were selected with an average initial body weight of 469.0 ± 15.8 kg (mean ± SD). In this case the number of cows in treatment was one (1) since the design was a 4 × 4 double “change-over design” set as Latin Squares in which individual animals can get each treatment. This all indicated in methodology except some modifications.

2. Table 1, 2, and 3 are my Ph.D result which was published in Asian Journal of Dairy and Food Research 2020. This work is my original work that helps the base line information to carry out feeding trial on those deficient minerals which were identified as a deficient, which dominantly affect the milk yield of lactating cows. Therefore, my previous result specially identification of deficient minerals which affect our dairy cows were identified from on farm lactating dairy feeds, dairy blood(serum) and finally this makes to made feeding trial (mineral) to what level supplementation give optimum milk production in our context. That’s why I used this data as a preliminary information as a base for my feeding trial.

3. Another comment is about analysis: SAS is software, and the result is analyzed through analysis of variance (ANOVA), mean minerals concentrations of samples were compared with General Liner Model (GLM) procedure that why I used however, I made some rephrasing.

NB: The rest are comments I accept and incorporate all.

- **Reviewer2.**
- The second reviewer has more focused on references:
- The total experimental period was consisted of four periods; the length of each period was 30 days (15days for adaptation and 15 days for data measurement) throughout 120 days…..page 5….. There is contradiction with abstract part… DCP was supplemented for 120 days of feeding trial period classified into four periods, and each consisting of 30 days (16 days of adaptation and 14 days of data collection) and at different levels (0; 0.3; 0.6; 0.9 % DM/day of DCP) for T0, T1, T2, T3, respectively.
- We accepted as a comment because there was different number in adaptation and data measurement the exact is 16 days for adaptation and 14 days for measurement period
- Except Endale et al., 2015 & Lemma et. al , 2016, the rest sources which are mentioned by second reviewer are available in the document. We have accepted and change

NB: finally, in case of proof reading and about the sponsorship

- We have accepted and made a change specially some redundant words, absence and presence as well as improper utilization of punctuations and rephrasing some phrases in result and discussion part
- The question have raised about the funding source, when I performed this research I was working my Ph.D in Adis Abeba University almost all budget from Adis Abeba university my Dilla University through the thematic project of Dr Tilaye Demissie who is included in publication (Advisor). However, the AGP-II (Agricultural Growth Program) through Debreziet Agricultural research allowed for us the dairy cow farm to perform our feeding trial simply because the Debrezeit Agricultural research need the output of the research and we did that as recommendation.
